# Reconstructing spleno‐mesenterico‐portal cofluence by bifurcated allogeneic vein in local advanced pancreatic cancer—a feasible method to avoid left‐sided portal hypertension

**DOI:** 10.1002/cam4.4093

**Published:** 2021-06-30

**Authors:** Xingmao Zhang, Qiao Wu, Hua Fan, Qiang He, Ren Lang

**Affiliations:** ^1^ Department of hepatobiliary surgery Beijing Chaoyang Hospital Capital Medical University Beijing China

**Keywords:** left**‐**sided portal hypertension, outcome, pancreatic cancer, reconstruction

## Abstract

**Background:**

Left‐sided portal hypertension is usually found in patients undergoing pancreaticoduodenectomy (PD) with spleno‐mesenterico‐portal (S‐M‐P) confluence resection. This study is to explore the outcomes of S‐M‐P confluence reconstruction after resection by using bifurcated allogeneic vein.

**Methods:**

Clinicopathologic data of patients who underwent extensive PD with S‐M‐P confluence resection for carcinoma of pancreatic head/uncinate process in our hospital between December 2011 and August 2018 were retrospectively reviewed and clinical outcomes of vein reconstruction after resection were analyzed.

**Results:**

Of the 37 patients enrolled, S‐M‐P reconstruction by bifurcated allogeneic vein was performed in 24 cases (group 1) and simply splenic vein ligation in 13 cases (group 2). Items including pathological results, blood loss, and complications were comparable between the two groups, operation time was longer in group 1 (573.8 vs. 479.2 min, *p *= 0.018). Significantly decreased platelet count (205.9 vs. 133.1 × 10^9^/L, *p *= 0.001) and increased splenic volume (270.9 vs. 452.2 ml, *p *< 0.001) were observed in group 2 at 6 months after operation. The mean splenic hypertrophy ratio was 1.06 in group 1 and 1.63 in group 2, respectively (*p *< 0.001). There were four patients with varices were found in group 2, none in group 1.

**Conclusions:**

Without increased complications, reconstructing S‐M‐P confluence by bifurcated allogeneic vein after resection may help to avoid left‐sided portal hypertension.

## 1 INTRODUCTION

Early invasion of portal venous system is one of the characteristics of pancreatic head/ uncinate process carcinoma.^1^, ^2^ Extensive pancreaticoduodenectomy (PD) with PV/SMV resection and reconstruction has been adopted by some surgeons although no consensus has been achieved.^3^, ^4^ Improved surgical outcomes may be provided by extensive resection.^5^ Invasion of spleno‐mesenterico‐portal (S‐M‐P) confluence is deemed as a tricky problem.^6^ Improper vascular management during operative procedure is associated with an increased incidence of postoperative complications. PV/SMV reconstruction with simply ligation of splenic vein (SV) is widely adopted after resection of S‐M‐P confluence. However, left‐sided portal hypertension, as one of the major complications, is frequently observed in patients without reconstruction of SV.

As a localized form of portal hypertension, left‐sided portal hypertension which is also referred to as sinistral, segmental, regional, localized, compartmental, lineal, or splenoportal hypertension is resulted from isolated obstruction of SV frequently.^7^ Left‐sided portal hypertension can lead to various harms including splenomegaly, hypersplenism, varices, and gastrointestinal bleeding, and patients’ health and quality of life is threatened by these harms seriously.^8^, ^9^

With the aim of avoiding left‐sided portal hypertension, several strategies have been carried out during operation procedures. For instance, simultaneous ligation of splenic artery, SV‐SMV anastomosis, SV‐inferior mesenteric vein (IMV) anastomosis, temporary mesocaval shunt with distal splenorenal shunt, and so forth.^10^, ^11^, ^12^, ^13^ We have been conducting an innovation method to deal with pancreatic head/uncinate process cancer with infiltration of S‐M‐P confluence from 2011 to now. After completion of PD with an “en‐bloc” resection of the S‐M‐P confluence, bifurcated allogeneic vein which was from donation after cardiac death was used for the reconstruction of confluence. Few reports have been touched upon this method, and the correlation between this method and left‐sided portal hypertension has not yet been analyzed.

## 2 MATERIALS AND METHODS

### 2.1 Perioperative management

All data of patients who underwent PD for carcinoma of pancreatic head/uncinate process between December 2011 and August 2018 at author's institution were collected and analyzed in this retrospective study. The protocol was approved by the Ethics Committee of our hospital. Blood examinations including tumor markers, liver and kidney function, coagulation function, and blood routine, etc. were provided before operation, abdominal contrast–enhanced multidetector‐row computed tomography (CT), and thoracic CT were used to exclude distant metastatic disease, pulmonary function test, and ultrasonic cardiogram were also performed for patients with the age >60 years. Patient demographics, perioperative, and postoperative details and clinicopathological factors were retrospectively collected from patient charts.

After operation, Doppler B‐ultrasound was used for observing the venous blood flow on the third and seventh day, and computed tomographic angiography (CTA) was used for evaluating the venous condition at one month. Physical examination and routine laboratory tests were performed monthly for each patient, thoracic CT and abdominal thin‐sliced (1–2 mm) contrast‐enhanced CT were carried out every 6 months during the first 2 years, and CT or other examinations were performed for patients before the routine CT scan at 6 months in case of abnormalities which were detected at the monthly follow‐up.

### 2.2 Surgical details

**FIGURE 1:**
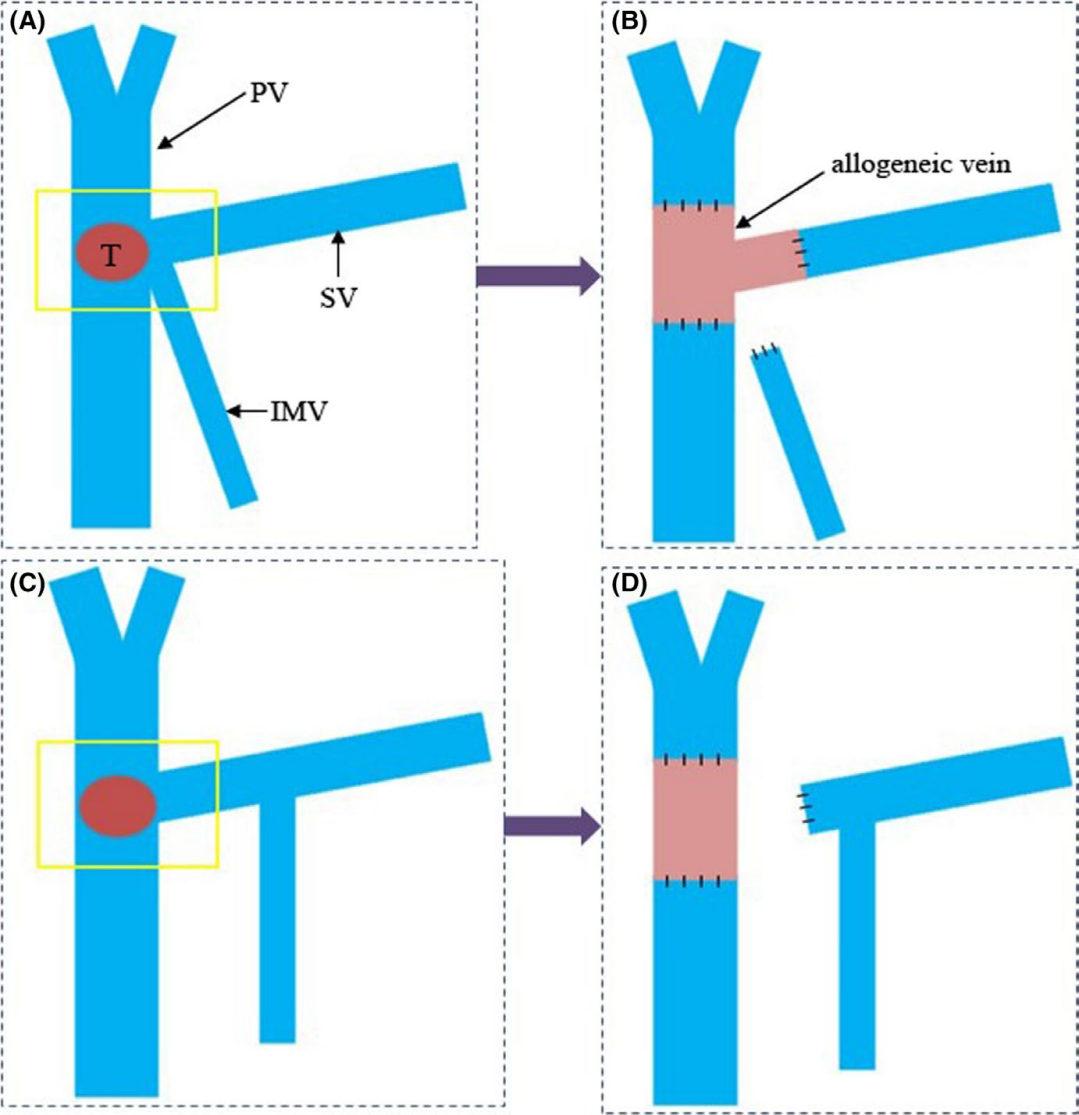
The operation procedures. Confluence was resected and splenic vein (SV) was cut off approximately 1–3 cm to the left of its confluence with superior mesenteric vein (SMV). The “T” meant tumor and the yellow square showed the resection range of portal vein system. *a–b*. Bifurcated allogeneic vein was used for reconstruction of spleno‐mesenterico‐portal confluence when inferior mesenteric vein (IMV) drained into SMV or splenomesenteric angle. *c–d*. SV was simply ligated when IMV drained into SV and natural SV‐IMV confluence could be preserved

Exploratory laparotomy was undertaken, and PD with extended lymphadenectomy including the hepatic hilum, common hepatic artery, celiac trunk, SMA, and para‐aortic area was carried out for patients with carcinoma of pancreatic head/uncinate process in the absence of metastases and dissemination. Venous resection and reconstruction were provided for patients with PV/SMV invasion. When invasion of S‐M‐P confluence was confirmed, confluence was resected and the SV was cut off approximately 1–3 cm to the left of its confluence with the SMV. The choice of reconstruction types was decided by the anatomy of SV: (a) Bifurcated allogeneic vein was used for reconstruction of S‐M‐P confluence when IMV drained into SMV or splenomesenteric angle. Splenic venous reflux was obstructed completely after resection of S‐M‐P confluence and only reconstruction of portal vein and SMV. (b) SV was simply ligated when IMV drained into SV and natural SV‐IMV confluence could be preserved. Some previous study showed that a natural SV‐IMV confluence could provide sufficient venous drainage of the spleen and gastric remnant.^14^, ^15^ According to the length of vein resected, either direct anastomosis or using allogeneic vein to reconstruct between PV and SMV should be selected. (Figure 1). The bifurcated allogeneic vein was from organ donor, the iliac vein, and sometimes the portal vein system of donor was preserved during trimming the donor liver.

**FIGURE 2:**
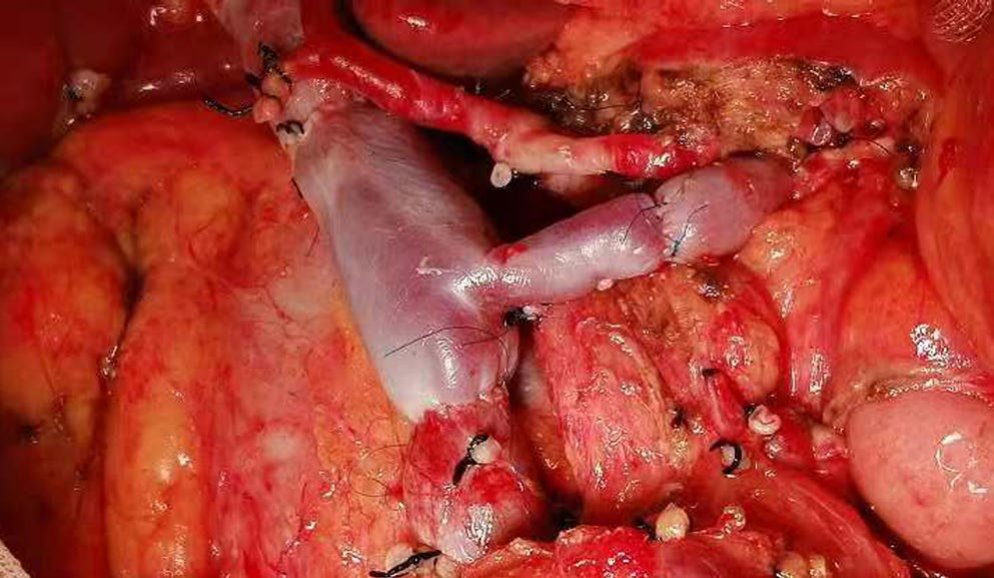
The completed anastomosis of S‐M‐P confluence

Procedures of anastomosis were as follows: anastomosis between stump of SMV and one end of allogeneic vein was performed first using a prolene purse string or vascular coupler device. The next step was to make the anastomosis between stump of PV and another one end of allogeneic vein. The anastomosis between the last end of allogeneic vein and stump of SV was started after restoring the blood supply of PV/SMV (Figure 2).

### 2.3 Surgical outcomes

Surgical outcomes, including intraoperative blood loss, operation time, curative resection (R0), postoperative complication, and so forth, were collected. R0 was defined as a specimen with clear resection margins, tumor cell was not found within 1 mm distance from margin. Postoperative complications were graded according to the Clavien–Dindo classification.^16^ Drain amylase of >3 times serum amylase after the third postoperative day, as defined by ISGPS (International Study Group of Pancreatic Surgery), was defined as pancreatic fistula.^17^

### 2.4 Splenic hypertrophy ratio

Total spleen volume was estimated by tracing the spleen on each transverse CT image obtained at 2.0‐mm intervals. Spleen volume was measured before operation and at 6 and 12 months after operation, respectively. Splenic hypertrophy ratio was calculated as post‐operation volume/pre‐operation volume. The existence of intra‐abdominal varices was evaluated at 3–6 months after operation by using enhanced CT.

### 2.5 Statistical analysis

SPSS 16.0 (IBM, Chicago, Illinois, USA) was used for data analysis. A value of *p* < 0.05 was considered to be statistically significant. Categorical variables were analyzed by chi‐square test, and continuous variables were analyzed by the Student's *t*‐test.

## 3 RESULTS

**FIGURE 3:**
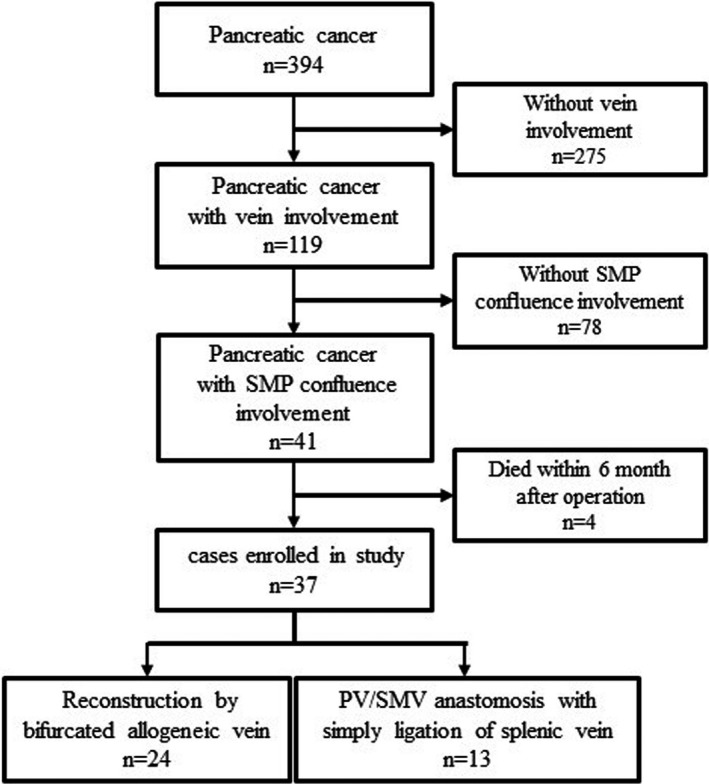
The patients flow in this study

Data of 394 patients who underwent PD during the study interval were reviewed. Of these, a total of 41 patients underwent excision of S‐M‐P confluence. Excepting four patients who died within 6 months after operation, the remaining 37 patients were enrolled in this study (Figure 3). Among the 37 patients, confluence reconstruction by using bifurcated allogeneic vein was adopted in 24 cases (group 1) and PV/SMV reconstruction with SV simply ligation in 13 cases (group 2).

General parameters of patients and postoperative pathology between the two groups were comparable (Tables 1 and 2). Percutaneous transhepatic cholangial drainage (PTCD) was used for patients who had the indication of preoperative biliary drainage. Six patients received neoadjuvant therapy in group 1 (mFOLFIRINOX for four cases, gemcitabine plus albumin‐bound paclitaxel for two cases), and four patients in group two (mFOLFIRINOX for three cases, gemcitabine plus albumin‐bound paclitaxel for one cases). No operation‐related deaths were found in the two groups. As shown in Table 2, operation time was significantly longer in group one compared to group two (573.8 vs. 479.2 min, *p* = 0.018); intraoperative blood loss was 657.9 ml in group 1 and 598.5 ml in group 2, no significant difference was revealed (*p* = 0.476). Incidences of total complications were similar between the two groups (33.3 vs. 38.5%, *p* = 0.755), meanwhile, no significant difference was found in incidence of pancreatic fistula (20.8 vs. 23.1%, *p* = 0.874). There was no obvious difference in Clavien‐Dindo classification of complications between the two groups (*p* = 0.962). No vascular‐related complications including venous thrombus and infection during the hospital stay and the follow‐up period were observed.

**TABLE 1:** General parameters of patients between two groups

Parameters	Group 1 (n = 24)	Group 2 (n = 13)	*p*‐values
Gender			0.666
Male	13	8	
Female	11	5	
Age, years			0.793
<60	10	6	
≥60	14	7	
ASA score			0.371
2	9	3	
3	15	10	
BMI, Kg/M^2^			0.616
<18.5	6	1	
18.5–24.9	13	8	
25–30	4	3	
>30	1	1	
Concomitant disease			0.482
Yes	15	10	
No	7	3	
Back pain			0.755
Yes	8	5	
No	16	8	
Preoperative biliary drainage			0.690
Yes	7	3	
No	17	10	
Elevated CA199 level			0.920
Yes	20	11	
No	4	2	
Neoadjuvant therapy			0.706
Yes	6	4	
No	18	9	
Platelet count, ×10^9^/L	222.8	239.4	0.474
Splenic volume, mL	255.9	275.3	0.145

Abbreviations: ASA, American Society of Anesthesiologists; BMI, Body Mass Index.

**TABLE 2:** Intraoperative and postoperative outcomes between two groups

Parameters	Group 1 (n = 24)	Group 2 (n = 13)	*p*‐values
Operation time, mean, min	573.8	479.2	0.018
Intraoperative blood loss, mean, ml	657.9	598.5	0.476
Blood transfusion, cases (yes/no)	8/16	3/10	0.515
Tumor location, cases (head/ uncinate)	20/4	11/2	0.920
Tumor size, mean, cm	3.7	3.6	0.915
Tumor differentiation, cases (well/moderate/poor)	4/6/14	2/5/6	0.686
Pathological type, cases (adenocarcinoma/others)	21/3	12/1	0.653
Perineural invasion, cases (yes/no)	21/3	11/2	0.862
Lymph nodes retrieved, mean	24.8	23.3	0.583
Lymph nodes involved, mean	5.0	4.4	0.626
TNM stage, cases (Ⅰb/Ⅱa/Ⅱb/Ⅲ)	1/1/9/13	0/1/5/7	0.864
Length of vein resected, mean, cm	2.6	2.8	0.492
Resection margin, cases (R0/R1)	19/5	10/3	0.874
Total complications, cases (yes/no)	8/16	5/8	0.755
Pancreatic fistula, cases (yes/no)	5/19	3/10	0.874
Clavien‐Dindo classification of complications (Ⅰ/Ⅱ/Ⅲa), cases	2/6/3	1/4/1	0.962
Platelet count at 6 months postoperatively, ×10^9^/L	205.9	133.1	0.001
Splenic volume at 6 months postoperatively, mean, mL	270.9	452.2	<0.001
splenic hypertrophy ratio at 6 months postoperatively, mean	1.06	1.63	<0.001

There was no obvious difference in the platelet count between the two group before operation (222.8 vs. 239.4×10^9^/L, *p* = 0.474), whereas the platelet count was significantly lower in group 2 compared to that in group 1 at 6 months after operation (205.9 vs. 133.1 × 10^9^/L, *p* = 0.001). The platelet count did not have significant change before and after operation in group 1 (222.8 vs. 205.9 × 10^9^/L, *p* = 0.376), in contrast, it decreased obviously after operation in group 2 (239.4 vs. 133.1 × 10^9^/L, *p* < 0.001).

**FIGURE 4:**
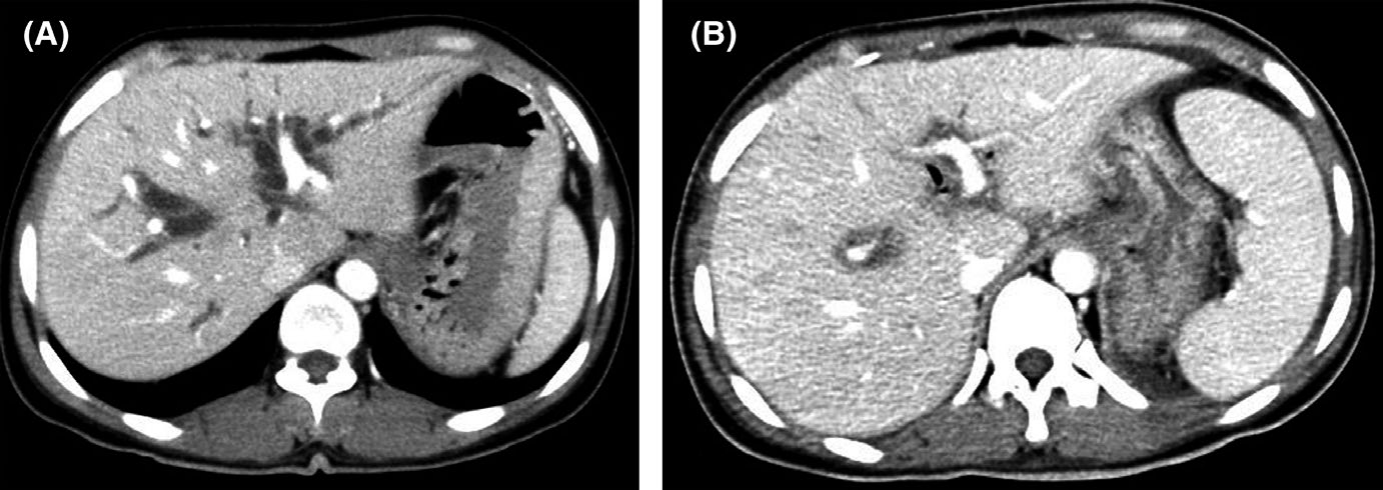
The change of splenic volume before and 6 months after operation on abdominal CT *a*. Splenic volume before operation, the maximum diameter was less than 5 rib units. *b*. Splenic volume increased significantly 6 months after operation, and the maximum diameter was more than 8 rib units

The mean splenic volume was comparable between group 1 and 2 before operation (255.9 vs. 275.3 mL, *p* = 0.145), and the splenic volume increased to 270.9 mL at 6 months after operation in group 1, but it increased to 452.2 mL in group 2, significantly statistical difference could be found (*p* < 0.001); the mean splenic hypertrophy ratio was 1.06 (0.99–1.30) in group 1 and 1.63 (1.03–2.13) in group 2, respectively (*p* < 0.001), shown in Figure 4. The mean splenic hypertrophy ratio was 1.06 (1.03–1.26) and 1.64 (1.07–2.10) at 12 months after operation in group 1 and 2, respectively, which was similar to that at 6 months after operation.

**FIGURE 5:**
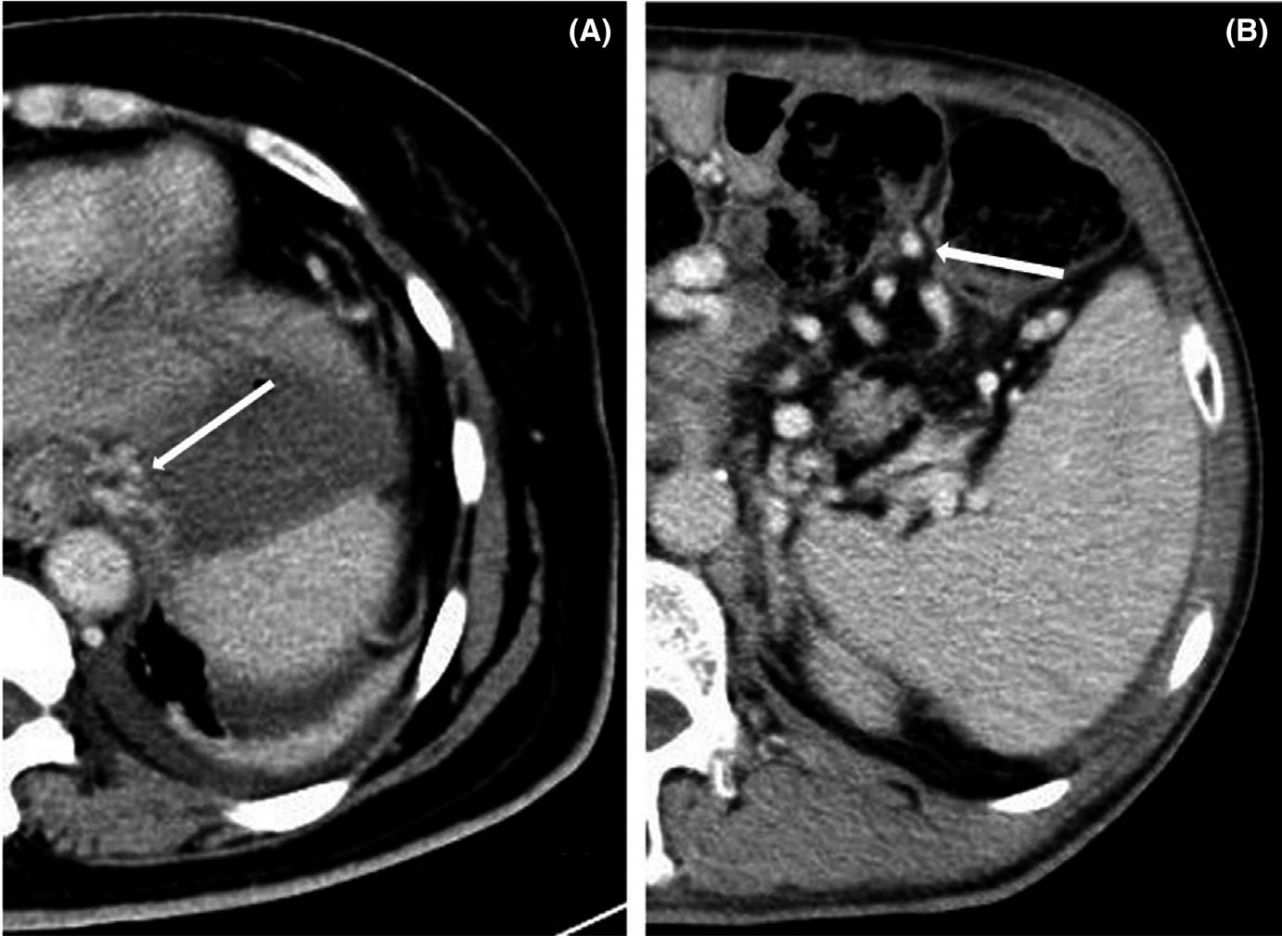
The varices detected during follow‐up period after operation. *a*. The white arrow showed esophageal and gastric varices. *b*. The white arrow showed colonic varices

There were no patients with varices in group 1 during the follow‐up period, but four patients with varices were confirmed by contrast‐enhanced CT in group 2, esophageal, and gastric varices was found in three patients and colonic varices in one patient (Figure 5).

## 4 DISCUSSION

With the aim of achieving the margin–free resection, PD with ‘‘en‐bloc’’ resection of the S‐M‐P confluence is currently performed for patients diagnosed with borderline resection or local advanced pancreatic cancer with infiltration of confluence.^12^, ^18^, ^19^ SV sometimes should be ligated during the operation procedures. Unfortunately, obstruction of venous reflux induced by ligation of SV may result in left‐sided portal hypertension. Although left‐sided portal hypertension is rare, it is known as a life‐threatening cause of upper gastrointestinal bleeding. Left‐sided portal hypertension has become a critical problem for patients with resection of S‐M‐P confluence till now.^20^

Several clinical issues and reconstruction techniques have been described after resection of S‐M‐P confluence.^20^, ^21^, ^22^, ^23^ Some surgeons reported that there was no need for reconstruction of the SV and there were no serious complications after SV ligation, for instance, Tanakaet al.^24^ suggested that PD with S‐M‐P confluence resection without SV reconstruction could be safely conducted, preservation of left gastric vein‐PV and/or IMV‐SV confluences might reduce the risk of left‐sided portal hypertension; whereas most surgeons had different views and they performed various reconstruction methods to avoid or lessen left‐sided portal hypertension.^9^, ^25^

Ferreira N and his colleagues^15^ reported that incidence of left‐sided portal hypertension could be significantly decreased by SV‐IMV anastomosis or preservation of the natural SV‐IMV confluence. However, Hattori M and his colleagues^26^ revealed that left‐sided portal hypertension could not be prevented by preserving SV‐IMV confluence. In this study, we performed simply ligation of SV when natural SV‐IMV confluence could be preserved. Our results showed that both of the platelet count and the splenic volume changed significantly at 6 months after operation compared to that before operation, meanwhile, a high incidence of varices of 30.8% was found. These results suggested that preservation of natural SV‐IMV confluence could not achieve the desired effect. Ono Y and his colleagues^9^ revealed that left–sided portal hypertension could not be avoided by SV‐IMV anastomosis or preservation of the natural SV‐IMV confluence because the blood flow from the spleen was the same when the IMV was divided.

Reconstructing S‐M‐P confluence by bifurcated allogeneic vein has its own advantages. The biggest advantage is that it is good for restoring normal anatomical structure and fluid dynamics to the greatest extent because of its natural bifurcations. We confirmed that venous reconstruction by allogeneic vein was feasible and safe through close observation. Compared with SV simply ligation, reconstruction by allogeneic vein had comparable intraoperative blood loss and no increased incidences of total complications and pancreatic fistula were observed although longer operation time was needed. Vascular‐related complications such as venous thrombus, infection, and graft rejection were not detected. In this study, we did not find left‐sided portal hypertension in patients underwent reconstruction by bifurcated allogeneic vein. Platelet count had no obvious change before and after operation and no patients with varices were found. The splenic hypertrophy ratio slightly increased at 6 months after operation in patients with reconstruction by bifurcated allogeneic vein, and unmatched vascular caliber might be the main cause for this phenomenon of slightly increased splenic hypertrophy ratio because we found that allogeneic veins with smaller caliber relative to their own were provided for a few patients.

The main limitation of the study is that the small number of patients enrolled may limit the accuracy of the final conclusion. Second, the period of follow‐up time was short and the long‐term outcomes are difficult to be observed. Future studies, preferably larger patient cohorts from multicenters, are needed to further confirm our preliminary outcomes.

## 5 CONCLUSIONS

Without increased postoperative complications, reconstructing S‐M‐P confluence by bifurcated allogeneic vein can help to avoid left‐sided portal hypertension. Reconstruction by bifurcated allogeneic vein may be a selected method to prevent left‐sided portal hypertension after resection of the S‐M‐P confluence in patients with pancreatic cancer.

## CONFLICT OF INTEREST

None

## AUTHOR CONTRIBUTIONS

(I) Conception and design: Xing‐mao Zhang and Qiang He; (II) Administrative support: Ren Lang; (III) Provision of study materials or patients: Hua Fan and Qiao Wu; (IV) Collection and assembly of data: Xing‐mao Zhang and Hua Fan; (V) Data analysis and interpretation: Qiao Wu and Ren Lang; (VI) Manuscript writing: All authors; (VII) Final approval of manuscript: All authors.

## ETHICAL STATEMENT

The study was conducted in accordance with the Declaration of Helsinki (as revised in 2013). The protocol was reviewed and approved by the institutional review committee of Beijing Chaoyang Hospital (approval number: 2020‐科‐304) and individual consent for this retrospective analysis was waived.

## Data Availability

The data that support the findings of this study are available from the corresponding author upon reasonable request.
